# A New Antibacterial Protocol Based on *Cymbopogon nardus* (L.) Rendle Essential Oil and UV-C Radiation with Potential Application for Hatching Eggs

**DOI:** 10.3390/antibiotics15080740

**Published:** 2026-07-31

**Authors:** Gabriel da Silva Oliveira, Concepta McManus, Fernanda Delgado, Joana Domingues, Josemar Gonçalves de Oliveira Filho, Cristiane Batista Salgado, Heloisa Alves de Figueiredo Sousa, Vinícius Machado dos Santos

**Affiliations:** 1 Laboratory of Poultry Science, Federal Institute of Brasília—Campus Planaltina, Brasília 73380-900, Brazil; 2 Faculty of Veterinary Medicine and Animal Science, University of São Paulo, São Paulo 05508-270, Brazil; 3 Faculty of Agronomy and Veterinary Medicine, University of Brasília, Brasília 70910-900, Brazil; 4 Center for Nuclear Energy in Agriculture (CENA), University of São Paulo, São Paulo 13416-000, Brazil; 5 School of Agriculture, Polytechnic Institute of Castelo Branco (ESA-IPCB), 6001-909 Castelo Branco, Portugal; 6 Research Centre for Natural Resources, Environment and Society, Polytechnic Institute of Castelo Branco (CERNAS-IPCB), 6001-909 Castelo Branco, Portugal; 7 Plant Biotechnology Centre of Beira Interior (CBPBI), 6001-909 Castelo Branco, Portugal; 8 Health Sciences Research Centre (CICS), University of Beira Interior, 6200-506 Covilhã, Portugal; 9 Embrapa Instrumentation, Brazilian Agricultural Research Corporation, São Carlos 13561-206, Brazil; 10 Laboratory of Geosciences and Human Sciences, Federal Institute of Brasília—Campus Brasília, Brasília 70830-450, Brazil; 11 Laboratory of Microbiology and Food, Federal Institute of Brasília—Campus Planaltina, Brasília 73380-900, Brazil

**Keywords:** eggshell quality, egg sanitization, natural products, poultry microbiology

## Abstract

**Background/Objectives:** Hatching egg sanitization protocols are primarily developed to protect embryos during the embryonic stage from exposure to horizontally transmitted pathogenic bacteria, thereby ensuring their survival. This study aimed to evaluate the antibacterial efficacy of *Cymbopogon nardus* (L.) Rendle (Poaceae) essential oil, alone or combined with UV-C radiation, as a potential sanitization protocol for hatching eggs. **Methods:** Unwashed table eggs, used as an experimental model, were sprayed with *C*. *nardus* essential oil at two concentrations (0.625–1.25%) and exposed to UV-C radiation for different exposure times (0–10 min). Mesophilic bacterial load and the physical characteristics of the eggshell were subsequently evaluated. **Results:** A significant reduction in the mesophilic bacterial load on the eggshell surface was observed following the application of the treatments. *C. nardus* essential oil promoted reductions greater than 2 log_10_ CFU/mL, demonstrating strong antimicrobial activity. The application of UV-C radiation alone resulted in a progressive decrease in the bacterial population, reaching approximately 2 log_10_ CFU/mL after 10 min of exposure. However, the combination of the essential oil and UV-C radiation showed the highest antibacterial efficacy, reducing bacterial counts to below 1 log_10_ CFU/mL, indicating that the combination of the two technologies was more effective than either treatment applied alone. No significant macroscopic changes were observed in the physical characteristics of the eggshells. **Conclusions:** The combined application of *C. nardus* essential oil and UV-C radiation on the eggshell surface may represent a promising sanitization protocol with potential application for hatching eggs.

## 1. Introduction

A proportion of chicken embryos die during the incubation process as a result of bacterial contamination. Bacteria belonging to genera such as *Salmonella*, *Escherichia*, *Staphylococcus*, and *Pseudomonas*, among others, have been isolated from dead embryos, demonstrating the ability of these microorganisms to contribute to embryonic mortality [1]. The bacteria may have originated from bacterial populations that proliferated on the eggshell surface, underscoring the need for preventive antibacterial measures, such as sanitizing hatching eggs. However, formaldehyde remains the most widely used intervention for this purpose, despite not providing an ideal balance between antibacterial efficacy and embryonic safety, as its toxic effects on embryos have already been demonstrated [2]. Furthermore, it has potential toxicity to human health, especially in commercial poultry production environments. It is recommended that indoor formaldehyde concentrations should not exceed 0.1 mg/m^3^ (30 min average) to prevent adverse health effects [3], including bodily irritation, respiratory disorders, and cancer [4]. However, hatching egg fumigation is commonly performed using much higher formaldehyde concentrations (e.g., 5 g/m^3^) [2]. Therefore, the search for effective antibacterial alternatives with lower toxicity than formaldehyde remains ongoing.

Ultraviolet radiation is another approach for controlling bacteria on eggshell surfaces, with different application protocols being described and continuously evaluated, generally using the 254 nm wavelength of the electromagnetic spectrum (UV-C radiation) [5,6,7,8,9]. In 2019, a UV-C radiation protocol (254 nm), applied at an average intensity of 8.09 mW/cm^2^ for 120 s, significantly reduced the total aerobic bacterial count on the eggshell surface from 2.97 ± 0.25 log_10_ CFU/mL (dry control) to 1.64 ± 0.21 log_10_ CFU/mL, showing efficacy similar to that observed for formaldehyde (1.85 ± 0.27 log_10_ CFU/mL) [10]. Yu et al. [11] demonstrated that UV-C radiation can synergize with chemical products, enhancing antibacterial activity on eggshell surfaces.

Essential oils are volatile compounds extracted from aromatic plants and have been tested and recommended for the control of bacteria on eggshell surfaces [12,13]. The essential oil extracted from the leaves of *Cymbopogon nardus* (L.) Rendle (Poaceae), commonly known as citronella, is one such example. It is generally a yellow to pale-yellow antibacterial oil, and its chemical composition is variable, as reported across different studies. For example, de Silva et al. [14] identified more than 25 chemical compounds, with geraniol, DL-limonene, citronellal, β-citronellol, geranyl acetate, and cis-ocimene as the major constituents. When sprayed onto the surface of eggs, *C. nardus* leaf essential oil reduced total aerobic mesophilic bacterial counts by approximately 4 log_10_ CFU/mL and Enterobacteriaceae to levels below 1 log_10_ CFU/mL [15].

UV-C radiation has been shown to enhance the antibacterial activity of essential oils, reducing bacterial contamination on egg surfaces, suggesting a possible synergistic interaction [16]. Although *C. nardus* essential oil has previously been shown to be effective in reducing bacterial contamination on eggshells [15], to the best of our knowledge, no studies have evaluated its combined application with UV-C radiation for egg sanitization. *C. nardus* essential oil is characterized by major constituents with potent antibacterial activity, such as geraniol, which acts by increasing bacterial membrane permeability [17]. Considering that UV-C radiation exerts its antibacterial action mainly through damage to bacterial DNA [18], and that the major constituents of *C. nardus* essential oil are capable of promoting structural alterations in bacterial cells, including increased membrane permeability, it is assumed that these compounds may increase bacterial susceptibility to the effects of UV-C radiation. Thus, we hypothesized that the combination of *C. nardus* essential oil and UV-C radiation would promote a greater reduction in bacterial load on eggshells than either treatment alone, and that this efficacy would depend on both the essential oil concentration and the duration of UV-C exposure. To test this hypothesis, the objective of this study was to develop and evaluate a new antibacterial protocol with potential application to hatching eggs based on the combined application of *C*. *nardus* essential oil and UV-C radiation. For this purpose, unwashed table eggs were used as an experimental model because the study focused exclusively on the bacteriological evaluation of the eggshell and the physical characteristics of the eggs, without investigating parameters related to embryonic development, hatchability or poultry performance.

## 2. Results and Discussion

### 2.1. Bacterial Count on Eggshells

Table 1 presents mesophilic bacterial counts recovered from eggshells subjected to spraying and/or UV-C radiation, revealing a significant interaction (*p* < 0.05) between these factors. The non-treated eggs (control group), which received neither liquid spraying nor UV-C radiation, exhibited the highest bacterial count, with a mean of 3.73 log_10_ CFU/mL. However, a progressive and significant reduction (*p* < 0.05) in bacterial counts was observed following UV-C exposure, reaching a mean of 1.64 log_10_ CFU/mL after 10 min. The antibacterial efficacy of UV-C radiation is attributed to its ability to induce DNA damage and disrupt bacterial replication and gene transcription processes [19,20]. Eggs sprayed exclusively with the essential oil-based sanitizer also showed significant reductions (*p* < 0.05), with mean bacterial counts below 1.5 log_10_ CFU/mL. This result may be associated with the antibacterial activity of geraniol and citronellal, two of the major constituents of *C. nardus* essential oil. Majewska-Smolarek and Kowalewska [21] reported that geraniol can exert antibacterial activity by increasing bacterial membrane permeability through interactions with membrane lipids, as well as by inhibiting quorum-sensing signaling and suppressing biofilm formation and bacterial adhesion. According to Kang et al. [22], citronellal alters the bacterial cell membrane upon contact, increasing extracellular leakage of nucleic acids, impairing energy metabolism, and interfering with DNA synthesis and repair. When eggs sprayed with the essential oil were subsequently exposed to UV-C radiation for 5 min or longer, bacterial counts decreased further (*p* < 0.05) to values below 1 log_10_ CFU/mL. Spraying with grain alcohol produced a significant effect (*p* < 0.05) only when combined with UV-C radiation, reducing the mean bacterial count from 3.18 to 1.58 log_10_ CFU/mL after 10 min of exposure. These findings indicate a potentiating effect of UV-C radiation on the antibacterial activity of the essential oil across all concentrations tested. This combined effect may be attributed to the antibacterial constituents of *C*. *nardus* essential oil, such as geraniol and citronellal, which compromise bacterial cell integrity, metabolic activity, and adaptive responses, thereby increasing bacterial susceptibility to UV-C-induced DNA damage. The absence of a significant effect of grain alcohol when applied alone further supports that the bacterial reduction observed in this treatment was predominantly associated with the action of UV-C radiation. This finding also indicates that the mechanical removal of non-adherent bacteria associated with the spraying procedure did not contribute significantly to the reduction in bacterial counts.

In this study, the composition of the eggshell microbiota was not characterized, and the persistence of the antibacterial effect over time was not evaluated. Although the observed reduction in mesophilic bacterial counts demonstrates the antibacterial potential of the proposed protocol, the identification of the affected bacterial taxa and the assessment of the persistence of this antibacterial effect over time would provide a more comprehensive evaluation of its efficacy. Such analyses would strengthen the evidence supporting the protocol and its potential application. Therefore, further studies addressing these issues are needed.

### 2.2. Physical Characteristics of Eggshells

Table 2 and Table 3 present the results of the physical characteristics of eggshells subjected to essential oil spraying and/or UV-C radiation exposure. No effects of the essential oil, UV-C radiation exposure, or the interaction between these factors were observed on the colorimetric parameters L*, a*, and b* (*p* > 0.05) (Table 2). The L* values remained similar across treatments, indicating no change in eggshell brightness. Likewise, the a* and b* parameters showed positive values in all evaluated groups, indicating the predominance of reddish and yellowish hues, characteristic of light brown eggshells, with no significant differences among treatments.

The absence of changes in the colorimetric parameters suggests that the application of *C. nardus* essential oil and UV-C radiation did not interfere with the surface pigmentation of the eggshells, which is important for preserving the visual perception of egg quality. Essential oils may influence eggshell coloration through naturally occurring pigmented compounds in their composition [23]. However, although *C. nardus* essential oil exhibited a pale-yellow coloration, its application alone, under the conditions evaluated in this study, was not sufficient to promote measurable changes in eggshell color, suggesting that such effects may depend on the application conditions of the essential oils.

No significant interaction between essential oil spraying and UV-C radiation was observed (*p* > 0.05) for egg weight (g), absolute and relative eggshell weight (g and %), eggshell weight per unit surface area (mg/cm^2^), or eggshell thickness (mm), indicating the absence of a combined treatment effect on the evaluated characteristics (Table 3). Additionally, when analyzed separately, neither spraying nor UV-C radiation exerted a significant influence (*p* > 0.05) on egg weight, absolute and relative eggshell weight, eggshell weight per unit surface area, or eggshell thickness. Similar results were reported by El-Soufi et al. [12], who evaluated the application of essential oils from *Syzygium aromaticum* (clove), *Cinnamomum verum* (cinnamon), *Mentha piperita* (peppermint), and *Salvia rosmarinus* (rosemary), as well as UV-C radiation, on egg quality during storage. The authors observed that reductions in egg weight and eggshell thickness occurred over time, but no significant differences were detected among treatments.

Relative eggshell weight and eggshell weight per unit surface area have been described as indicators of eggshell strength [24]. The absence of significant differences among these variables suggests that eggshells from the different treatments exhibit similar structural strength, indicating a comparable likelihood of cracking. Therefore, essential oils and UV-C radiation do not appear to promote significant macroscopic changes in the physical characteristics of eggs. In hatching eggs, maintaining these parameters is important because undesirable modifications may compromise embryonic protection, respiration, calcification, and thermoregulation [25,26].

### 2.3. Chemical Composition Analysis of C. nardus Essential Oil Before and After Exposure to UV-C Radiation

GC-MS analysis identified 99.8% of the constituents of *C. nardus* essential oil not exposed to UV-C radiation and 99.6% of the constituents of the essential oil after exposure to UV-C radiation for 5 min (Table 4). The chemical composition was predominantly oxygenated monoterpenes, especially geraniol, citronellal, citronellol, α-citral, and neral, which together accounted for most of the chromatographic area. Among these compounds, geraniol was the major constituent, accounting for 39.1 ± 0.3% in the non-exposed essential oil and 39.9 ± 0.0% after 5 min of UV-C exposure. Citronellal also remained one of the main compounds, with values of 27.7 ± 0.3% and 26.6 ± 0.1%, respectively. Citronellol showed a slight variation, increasing from 13.5 ± 0.2% to 13.9 ± 0.1%, whereas neral and α-citral remained virtually unchanged at 3.8 ± 0.0% and 5.1 ± 0.0–0.1%, respectively. Likewise, minor compounds such as limonene, linalool, isopulegol, decanal, citronellyl acetate, geranyl acetate, caryophyllene, γ-muurolene, δ-cadinene, and caryophyllene oxide showed very similar values under both evaluated conditions. Therefore, exposure to UV-C radiation for 5 min appears not to have caused relevant changes in the volatile fraction detectable by GC-MS.

In a recent study conducted by Ramadhan et al. [28], geraniol (70.65%) was also identified as the major constituent of *C. nardus* essential oil. The authors additionally reported the presence of compounds such as citronellal, citronellol, limonene, linalool, neral, geranyl acetate, isopulegol, caryophyllene, and δ-cadinene, which were similar to those identified in the present study, although in different proportions. Environmental conditions, the characteristics of the source plant, and factors related to water availability and light incidence during cultivation may have contributed to the quantitative differences in volatile compounds [29].

The maintenance of the major compounds after UV-C irradiation helps explain the high antibacterial efficacy observed when the essential oil was combined with UV-C. Since geraniol, citronellal, and citronellol remained in similar proportions after treatment, it is likely that the antibacterial effect of the combination did not depend on significant chemical alterations of the essential oil, but rather on the combined action of the intrinsic antimicrobial activity of these compounds and the bacterial damage promoted by UV-C radiation, as mentioned previously. Furthermore, the relative preservation of the essential oil’s chemical composition may be associated with the absence of macroscopic changes observed in the physical characteristics of the eggshells following application of the evaluated protocol.

Considering that it has previously been reported that the chemical composition of essential oils may be altered following exposure to UV-C radiation [30], the results observed in the present study suggest that the effects of UV-C radiation on the chemical composition of essential oils may depend on factors such as exposure time, distance from the radiation source, and the chemical composition of the essential oil being evaluated [30].

## 3. Materials and Methods

### 3.1. Obtaining C. nardus Essential Oil

*C. nardus* essential oil was commercially obtained from Laszlo (Belo Horizonte, Minas Gerais, Brazil) and stored under refrigeration (approximately 4 °C) until use. The essential oil was extracted from the plant’s leaves by steam distillation and had a density of 0.891 g/mL and a refractive index of 1.472 at 20 °C.

### 3.2. Minimum Inhibitory Concentration of C. nardus Essential Oil

The in vitro antibacterial activity of *C. nardus* essential oil was evaluated against *Staphylococcus aureus* ATCC 6538 and *Escherichia coli* ATCC 700926 using the broth microdilution method to assist in selecting the concentrations of the essential oil solutions to be applied to the eggs. The essential oil was emulsified with 0.05% (*v*/*v*) Tween 80, and serial dilutions of the essential oil (1.25–0.010%) were prepared in Mueller–Hinton broth. A single bacterial colony was inoculated into 25 mL of Brain Heart Infusion broth and incubated at 37 ± 2 °C for 18–24 h. The resulting bacterial suspensions were adjusted to approximately 10^6^ CFU/mL, and 100 μL was transferred to the wells of a 96-well microplate, which was then incubated at 37 °C for 24 h. After incubation, 10 μL of a 1% 2,3,5-triphenyl tetrazolium chloride solution was added to each well, and the plates were incubated for an additional 1 h at 37 ± 2 °C. The minimum inhibitory concentration (MIC) was determined as the lowest concentration of essential oil that produced no color change, indicating complete inhibition of bacterial growth [31].

The results of this assay demonstrated that the MIC of *C. nardus* essential oil against *Staphylococcus aureus* and *Escherichia coli* was 0.313 and 0.625%, respectively (Table 5). As the lowest concentration capable of inhibiting both bacteria was 0.625%, this concentration was selected for preparing the sanitizing solutions. A concentration of 1.25% was also evaluated, given that the MIC may not exhibit the same efficacy in vivo and to determine whether a higher essential oil concentration could provide greater antibacterial effectiveness.

### 3.3. Experimental Design and Application of C. nardus Essential Oil and UV-C Radiation on Eggs

**Figure 1:**
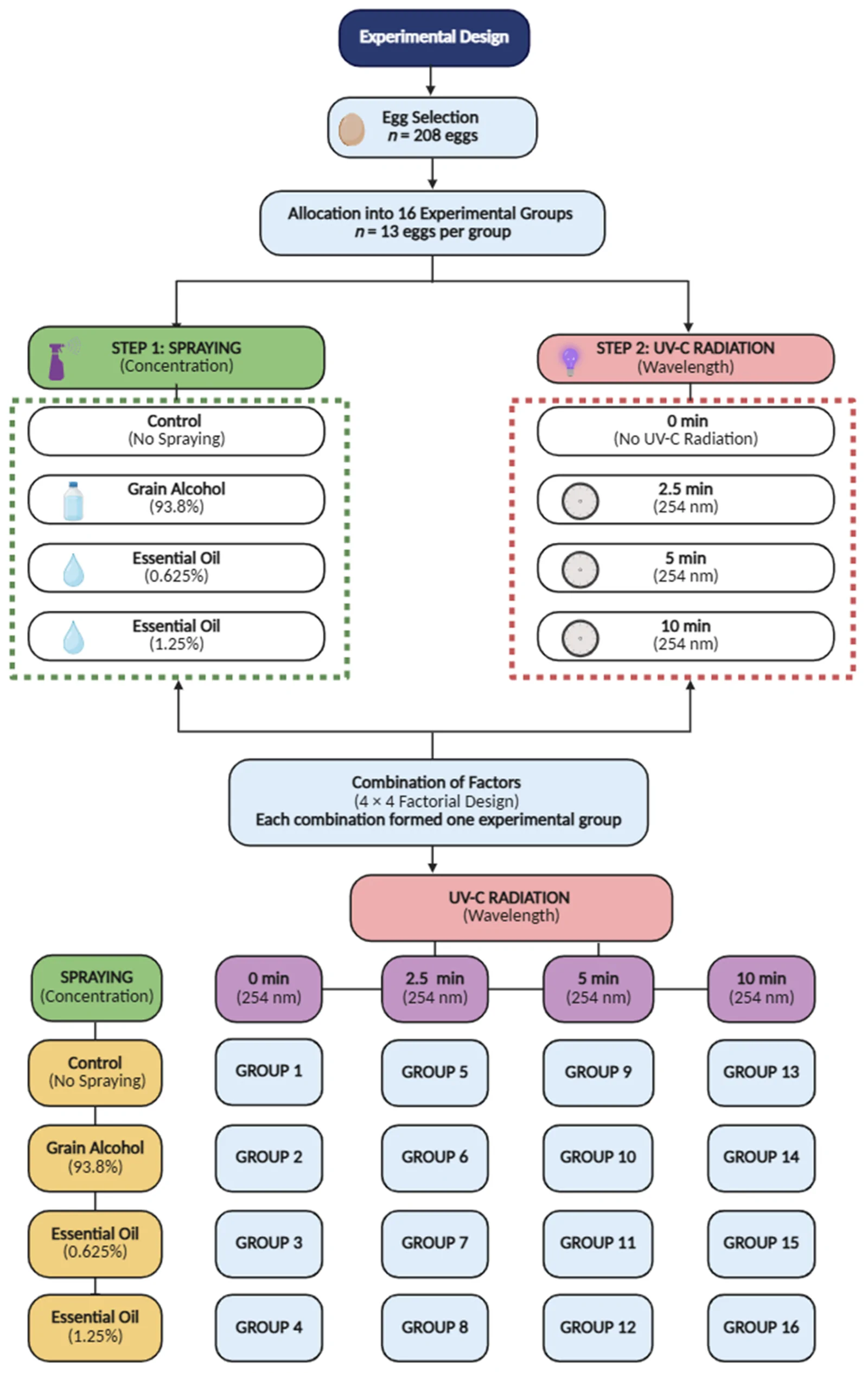
Experimental design of the evaluated treatments.

This study examined the effects of *C. nardus* essential oil and UV-C radiation on eggs using a factorial arrangement in a completely randomized design. The first factor was spraying, with a control group (no spraying), spraying with 93.8% grain alcohol (the diluent used for the essential oil), and spraying with essential oil at 0.625 and 1.25%. The second factor was UV-C radiation, applied at four exposure times: 0 (control), 2.5, 5, and 10 min. Thus, the experiment consisted of 16 treatments resulting from the combination of the levels of both factors, with 13 eggs per treatment, for a total of 208 eggs (Figure 1).

Unwashed table eggs were collected directly from nest boxes in a cage-free system housing 54-week-old Embrapa 051 laying hens (Planaltina, Federal District, Brazil) and transported to the laboratory, where they were subjected to the treatments according to the experimental protocol of each group. Initially, the entire surface of each egg was individually sprayed with the essential oil (approximately 2 mL per egg) using a manual sprayer. After drying, the eggs were exposed to UV-C radiation using a laminar flow cabinet (Pachane, Line 300, Piracicaba, São Paulo, Brazil). The eggs were positioned horizontally on a rotating tray at 60 cm from the lamp and irradiated with UV-C (254 nm). Before each protocol, the UV-C lamp was turned on for 5 min to stabilize radiation output, and during the exposure, the eggs were manually rotated 3 times.

### 3.4. Bacterial Count on Eggshells

After the sanitization process, each egg was aseptically placed into a sterile bag containing 70 mL of 0.1% peptone saline solution, with three replicates performed per group. The eggshell surface was then gently rubbed for 2 min to remove bacteria. The resulting wash solutions were serially diluted, and mesophilic bacterial counts were determined by plating aliquots of each dilution onto Plate Count Agar (Laborclin, Pinhais, Paraná, Brazil) and incubating at 36 °C for 48 h. All bacterial counts were expressed as log_10_ CFU/mL.

### 3.5. Physical Characteristics of Eggshells

A total of 10 eggs per group were used to evaluate eggshell physical characteristics, with each egg considered an experimental replicate. The analyses included the determination of eggshell color at the center of the broad end of the egg using a portable colorimeter (Delta Color, São Leopoldo, Rio Grande do Sul, Brazil) to record the parameters L* (lightness), a* (redness), and b* (yellowness). Eggshell absolute weight (g), relative eggshell weight (%), eggshell weight per unit surface area (mg/cm^2^), and eggshell thickness (mm), including the shell membrane, measured at the equatorial plane using a precision digital caliper (Mitutoyo, Jundiaí, São Paulo, Brazil), were also evaluated. Eggshell weight per unit surface area was calculated as follows: [eggshell weight/(3.9782 × egg weight^0.7056^)] × 1000 [24].

### 3.6. Chemical Composition Analysis of C. nardus Essential Oil

The volatile profile of the essential oil was obtained, in triplicate, using gas chromatography coupled with mass spectrometry (GC/MS SCION-SQ 456 GC, Bruker, Billerica, MA, USA). The separation was performed on an HP-5MS capillary column (30 m × 0.25 mm i.d. × 0.25 µm film thickness, Agilent J&W, Folsom, CA, USA). Helium was used as the carrier gas at a flow rate of 1 mL/min. The diluted essential oil sample (1:100, 1 µL) was injected using a split ratio of 1:100 and analyzed by electron impact ionization mass spectrometry (EI-MS) at 70 eV. The compounds were identified in scan mode with positive ion polarity over a mass range of 20–300 *m*/*z* and a scan time of 250.0 ms. The initial oven temperature was set to 45 °C, increasing at a rate of 3 °C/min to 175 °C, then at 15 °C/min to 300 °C, where it was maintained for 10 min. The transfer line and ion source temperatures were set at 250 °C and 220 °C, respectively. Compound identification was based on retention indices (RIs) compared with those reported in the NIST 17 mass spectral library (version 2.3) and with RI values calculated from a homologous series of n-alkane standards (C7–C18 and C19–C30) injected under the same chromatographic conditions. The relative amount of each compound was expressed as the percentage of its peak area relative to the total area of all identified peaks in the sample [32].

### 3.7. Exposure of C. nardus Essential Oil to UV-C Radiation

Samples of *C. nardus* essential oil were placed in open glass vials and exposed to UV-C radiation under the same irradiation conditions used for egg sanitization. The exposure time was defined based on bacterial counts and evaluations of eggshell characteristics, aiming to select the most appropriate and effective exposure duration. After exposure, the samples were reanalyzed by GC–MS under the same analytical conditions previously described in order to assess possible changes in the chemical composition of the essential oil following UV-C irradiation.

### 3.8. Statistical Analysis

Data were subjected to analysis of variance (PROC GLM) to evaluate the effects of spraying treatment (control, grain alcohol, and essential oil at concentrations of 0.625 and 1.25%), UV-C radiation (0, 2.5, 5, and 10 min), and the interaction between these factors on bacterial counts and the physical characteristics of eggshells. Means were compared using Tukey’s test at the 5% significance level (*p* < 0.05). All statistical analyses were performed using SAS Studio version 9.4 University Edition (SAS Institute Inc., Cary, NC, USA), and the results were expressed as mean ± standard deviation.

## 4. Conclusions

In conclusion, this study demonstrated that applying *C*. *nardus* essential oil and UV-C radiation reduced the mesophilic bacterial load on the eggshell surface without causing significant macroscopic changes in its physical characteristics. The combined application of the essential oil, regardless of concentration, and UV-C radiation showed greater antibacterial efficacy than either treatment applied alone, demonstrating a potentiating effect of UV-C radiation on the essential oil’s antibacterial activity. Spraying the essential oil at a concentration of 0.625%, followed by exposure to UV-C radiation for 5 min, proved to be the most efficient sanitization strategy, as it corresponded to the lowest concentration and shortest exposure time tested to achieve the maximum bacterial reduction on the eggshell surface. Furthermore, exposure of the essential oil to UV-C radiation for 5 min did not induce detectable changes in its volatile chemical profile as measured by GC-MS, indicating the relative stability of the major compounds associated with its antibacterial activity. Additional studies using hatching eggs are required to validate the safety and efficacy of combining *C*. *nardus* essential oil at 0.625% with UV-C radiation for 5 min on hatchery performance, chick quality, and post-hatch performance, thereby supporting its practical application in poultry production.

## Figures and Tables

**Table 1:** Mesophilic bacterial counts on eggshells sprayed with different liquid solutions and subjected to different UV-C radiation exposure times.

Spraying Treatment	UV-C Radiation (min)			
	0	2.5	5	10
	Mesophilic Bacteria (log_10_ CFU/mL) ± Standard Deviation			
No Spraying	3.73 ± 0.18 ^A,a^	3.66 ± 0.24 ^A,a^	2.52 ± 0.15 ^A,b^	1.64 ± 0.27 ^A,c^
Grain Alcohol (93.8%)	3.18 ± 0.37 ^A,a^	2.99 ± 0.15 ^B,a^	1.93 ± 0.12 ^A,b^	1.58 ± 0.13 ^A,b^
*C. nardus* Essential Oil (0.625%)	1.23 ± 0.35 ^B,a^	1.31 ± 0.35 ^C,a^	<1.00 *^B,b^	<1.00 ^B,b^
*C. nardus* Essential Oil (1.25%)	1.37 ± 0.26 ^B,a^	1.11 ± 0.20 ^C,a^	<1.00 ^B,b^	<1.00 ^B,b^
*p*-value				
Spraying		<0.0001		
UV-C Radiation		<0.0001		
Spraying × UV-C Radiation		0.0166		

^A–C^; ^a–c^ Means followed by different uppercase letters within a column indicate significant differences among liquid treatments at the same UV-C radiation exposure time, whereas means followed by different lowercase letters within a row indicate significant differences among UV-C radiation exposure times within the same liquid treatment, according to Tukey’s test (*p* < 0.05). * The limit of detection was 1.00 log_10_ CFU/mL, corresponding to 10 CFU/mL. Treatments with bacterial counts below this limit were reported as <1.00 log_10_ CFU/mL.

**Table 2:** Eggshell color of eggs sprayed with different liquid solutions and subjected to different UV-C radiation exposure times.

Spraying Treatment	UV-C Radiation			
	0	2.5	5	10
	L*			
No Spraying	64.37 ± 3.02 ^A,a^	61.04 ± 3.62 ^A,a^	62.09 ± 3.96 ^A,a^	64.42 ± 4.94 ^A,a^
Grain Alcohol (93.8%)	61.38 ± 4.23 ^A,a^	62.44 ± 3.25 ^A,a^	61.19 ± 3.25 ^A,a^	63.78 ± 3.79 ^A,a^
*C. nardus* Essential Oil (0.625%)	62.20 ± 3.68 ^A,a^	62.99 ± 3.20 ^A,a^	64.75 ± 3.98 ^A,a^	64.85 ± 3.17 ^A,a^
*C. nardus* Essential Oil (1.25%)	63.02 ± 4.10 ^A,a^	62.94 ± 3.63 ^A,a^	64.04 ± 3.45 ^A,a^	61.67 ± 3.43 ^A,a^
*p*-value				
Spraying	0.3538			
UV-C Radiation	0.4346			
Spraying × UV-C Radiation	0.1996			
**Spraying Treatment**	**UV-C Radiation**			
	**0**	**2.5**	**5**	**10**
	a*			
No Spraying	13.19 ± 1.66 ^A,a^	15.32 ± 1.91 ^A,a^	15.16 ± 1.91 ^A,a^	13.12 ± 2.93 ^A,a^
Grain Alcohol (93.8%)	14.98 ± 2.13 ^A,a^	14.70 ± 1.77 ^A,a^	15.69 ± 2.17 ^A,a^	13.49 ± 2.23 ^A,a^
*C. nardus* Essential Oil (0.625%)	14.36 ± 2.54 ^A,a^	14.59 ± 1.97 ^A,a^	13.27 ± 2.08 ^A,a^	14.24 ± 2.28 ^A,a^
*C. nardus* Essential Oil (1.25%)	13.75 ± 2.46 ^A,a^	13.74 ± 2.19 ^A,a^	13.94 ± 2.04 ^A,a^	14.70 ± 1.52 ^A,a^
*p*-value				
Spraying	0.4803			
UV-C Radiation	0.3856			
Spraying × UV-C Radiation	0.0862			
**Spraying Treatment**	**UV-C Radiation (min)**			
	**0**	**2.5**	**5**	**10**
	b*			
No Spraying	25.76 ± 2.88 ^A,a^	27.27 ± 2.60 ^A,a^	27.23 ± 2.75 ^A,a^	24.34 ± 3.93 ^A,a^
Grain Alcohol (93.8%)	25.27 ± 2.99 ^A,a^	24.79 ± 3.09 ^A,a^	27.27 ± 3.46 ^A,a^	24.38 ± 3.61 ^A,a^
*C. nardus* Essential Oil (0.625%)	26.81 ± 2.46 ^A,a^	27.04 ± 2.68 ^A,a^	25.33 ± 3.12 ^A,a^	25.75 ± 2.91 ^A,a^
*C. nardus* Essential Oil (1.25%)	27.17 ± 2.69 ^A,a^	27.51 ± 2.44 ^A,a^	24.88 ± 1.65 ^A,a^	26.09 ± 1.52 ^A,a^
*p*-value				
Spraying	0.4364			
UV-C Radiation	0.1131			
Spraying × UV-C Radiation	0.1112			

^A^; ^a^ Means followed by the same uppercase letter within a column are not significantly different among liquid treatments at the same UV-C radiation exposure time, whereas means followed by the same lowercase letter within a row are not significantly different among UV-C radiation exposure times within the same liquid treatment, according to Tukey’s test (*p* > 0.05).

**Table 3:** Egg weight, absolute and relative eggshell weights, eggshell weight per unit surface area, and eggshell thickness of eggs sprayed with different liquid solutions and subjected to different UV-C radiation exposure times.

Spraying Treatment	UV-C Radiation (min)			
	0	2.5	5	10
	Egg Weight (g)			
No Spraying	58.49 ± 3.76 ^A,a^	57.70 ± 4.03 ^A,a^	57.97 ± 3.01 ^A,a^	58.96 ± 4.24 ^A,a^
Grain Alcohol (93.8%)	58.94 ± 3.03 ^A,a^	57.65 ± 3.58 ^A,a^	58.37 ± 4.46 ^A,a^	58.92 ± 4.60 ^A,a^
*C. nardus* Essential Oil (0.625%)	58.45 ± 3.63 ^A,a^	59.22 ± 3.50 ^A,a^	58.18 ± 3.39 ^A,a^	57.86 ± 4.00 ^A,a^
*C. nardus* Essential Oil (1.25%)	58.75 ± 3.09 ^A,a^	58.59 ± 4.68 ^A,a^	58.89 ± 4.30 ^A,a^	58.41 ± 3.63 ^A,a^
*p*-value				
Spraying	0.9777			
UV-C Radiation	0.9725			
Spraying × UV-C Radiation	0.9921			
**Spraying Treatment**	**UV-C Radiation (min)**			
	**0**	**2.5**	**5**	**10**
	**Absolute Eggshell Weight (g)**			
No Spraying	6.42 ± 0.65 ^A,a^	6.42 ± 0.62 ^A,a^	6.55 ± 0.58 ^A,a^	6.75 ± 0.48 ^A,a^
Grain Alcohol (93.8%)	6.90 ± 0.36 ^A,a^	6.34 ± 0.56 ^A,a^	6.51 ± 0.74 ^A,a^	6.55 ± 0.46 ^A,a^
*C. nardus* Essential Oil (0.625%)	6.50 ± 0.38 ^A,a^	6.46 ± 0.49 ^A,a^	6.32 ± 0.35 ^A,a^	6.63 ± 0.51 ^A,a^
*C. nardus* Essential Oil (1.25%)	6.58 ± 0.79 ^A,a^	6.83 ± 0.64 ^A,a^	6.71 ± 0.50 ^A,a^	6.50 ± 0.73 ^A,a^
*p*-value				
Spraying	0.5615			
UV-C Radiation	0.8185			
Spraying × UV-C Radiation	0.3662			
**Spraying Treatment**	**UV-C Radiation (min)**			
	**0**	**2.5**	**5**	**10**
	**Relative Eggshell Weight (%)**			
No Spraying	10.99 ± 1.01 ^A,a^	11.12 ± 0.69 ^A,a^	11.31 ± 0.98 ^A,a^	11.48 ± 0.87 ^A,a^
Grain Alcohol (93.8%)	11.72 ± 0.59 ^A,a^	11.01 ± 0.95 ^A,a^	11.15 ± 0.97 ^A,a^	11.17 ± 1.04 ^A,a^
*C. nardus* Essential Oil (0.625%)	11.13 ± 0.44 ^A,a^	10.91 ± 0.47 ^A,a^	10.88 ± 0.61 ^A,a^	11.47 ± 0.73 ^A,a^
*C. nardus* Essential Oil (1.25%)	11.18 ± 1.00 ^A,a^	11.65 ± 0.49 ^A,a^	11.42 ± 0.80 ^A,a^	11.11 ± 0.79 ^A,a^
*p*-value				
Spraying	0.5945			
UV-C Radiation	0.8712			
Spraying × UV-C Radiation	0.2061			
**Spraying Treatment**	**UV-C Radiation (min)**			
	**0**	**2.5**	**5**	**10**
	**Eggshell Weight per Unit Surface Area (mg/cm^2^)**			
No Spraying	91.45 ± 8.24 ^A,a^	92.24 ± 6.17 ^A,a^	93.90 ± 7.90 ^A,a^	95.71 ± 6.38 ^A,a^
Grain Alcohol (93.8%)	97.77 ± 4.39 ^A,a^	91.26 ± 7.51 ^A,a^	92.76 ± 8.38 ^A,a^	93.01 ± 7.40 ^A,a^
*C. nardus* Essential Oil (0.625%)	92.62 ± 3.35 ^A,a^	91.13 ± 4.40 ^A,a^	90.40 ± 4.46 ^A,a^	95.16 ± 5.76 ^A,a^
*C. nardus* Essential Oil (1.25%)	93.26 ± 8.97 ^A,a^	97.05 ± 4.86 ^A,a^	95.17 ± 6.01 ^A,a^	92.49 ± 7.50 ^A,a^
*p*-value				
Spraying	0.5240			
UV-C Radiation	0.8294			
Spraying × UV-C Radiation	0.1689			
**Spraying Treatment**	**UV-C Radiation (min)**			
	**0**	**2.5**	**5**	**10**
	**Eggshell Thickness (mm)**			
No Spraying	0.41 ± 0.02 ^A,a^	0.41 ± 0.01 ^A,a^	0.42 ± 0.03 ^A,a^	0.41 ± 0.03 ^A,a^
Grain Alcohol (93.8%)	0.40 ± 0.02 ^A,a^	0.41 ± 0.02 ^A,a^	0.41 ± 0.03 ^A,a^	0.40 ± 0.02 ^A,a^
*C. nardus* Essential Oil (0.625%)	0.42 ± 0.01 ^A,a^	0.42 ± 0.02 ^A,a^	0.43 ± 0.03 ^A,a^	0.40 ± 0.02 ^A,a^
*C. nardus* Essential Oil (1.25%)	0.40 ± 0.03 ^A,a^	0.40 ± 0.02 ^A,a^	0.41 ± 0.03 ^A,a^	0.40 ± 0.04 ^A,a^
*p*-value				
Spraying	0.0513			
UV-C Radiation	0.1036			
Spraying × UV-C Radiation	0.8325			

^A^; ^a^ Means followed by the same uppercase letter within a column are not significantly different among liquid treatments at the same UV-C radiation exposure time, whereas means followed by the same lowercase letter within a row are not significantly different among UV-C radiation exposure times within the same liquid treatment, according to Tukey’s test (*p* > 0.05).

**Table 4:** Identification, retention time (min), and relative amounts (%) of compounds present in *C. nardus* essential oil before and after exposure to UV-C radiation for 5 min.

Identified Compound	R.T. (min.)	RI_exp_	RI_lit_	Percentage in the Samples (%)	
				*C. nardus*	*C. nardus* + UV-C Radiation
6-Methyl-5-heptene-2-one	10.04	966	966	0.1 ± 0.0	0.1 ± 0.0
	10.13	968		0.0 ± 0.0	0.0 ± 0.0
Octanal	10.62	981	987	0.0 ± 0.0	0.0 ± 0.0
Limonene	11.66	1007	1028	1.9 ± 0.0	1.8 ± 0.1
	11.74	1008		-	0.0 ± 0.0
*trans*-β-Ocimene	12.18	1019	1039	0.2 ± 0.0	0.2 ± 0.0
β-Ocimene	12.63	1030	1043	0.1 ± 0.0	0.1 ± 0.0
2,6-Dimethyl-5-heptenal	12.89	1037	1044	0.2 ± 0.0	0.1 ± 0.0
4-Nonanone	13.76	1059	1053	0.3 ± 0.0	0.3 ± 0.0
Linalool	15.00	1089	1098	0.8 ± 0.0	0.8 ± 0.0
*trans*-Rose oxide	15.49	1101	1115	0.1 ± 0.0	0.1 ± 0.0
2,6-Dimethyl-5-hepten-1-ol	16.36	1123		0.1 ± 0.0	0.1 ± 0.0
Isopulegol	16.98	1139	1146	0.5 ± 0.0	0.4 ± 0.0
	17.21	1144		0.1 ± 0.0	0.1 ± 0.0
Citronellal	17.42	1149	1153	27.7 ± 0.3	26.6 ± 0.1
	17.94	1162		0.0 ± 0.0	0.0 ± 0.0
3,7-Dimethyl-3,6-octadienal	18.81	1184	1184	0.1 ± 0.0	0.1 ± 0.0
Decanal	19.84	1210	1207	0.5 ± 0.0	0.4 ± 0.0
Citronellol	20.88	1235	1232	13.5 ± 0.2	13.9 ± 0.1
Neral	21.41	1248	1248	3.8 ± 0.0	3.8 ± 0.0
**Geraniol**	**22.08**	**1265**	**1267**	**39.1 ± 0.3**	**39.9 ± 0.0**
α-Citral	22.76	1282	1287	5.1 ± 0.0	5.1 ± 0.1
	24.17	1317		-	0.0 ± 0.0
	25.42	1348		-	0.0 ± 0.0
Citronellol acetate	26.42	1373	1376	0.9 ± 0.0	0.9 ± 0.0
β-Bourbonene	27.62	1403	1384	0.0 ± 0.0	0.1 ± 0.0
Geranyl acetate	27.73	1405	1392	1.5 ± 0.0	1.6 ± 0.1
	27.97	1411		0.1 ± 0.0	0.1 ± 0.0
Caryophyllene	29.06	1438	1444	1.7 ± 0.0	1.7 ± 0.1
Humulene	30.46	1473	1477	0.2 ± 0.0	0.2 ± 0.0
Germacrene D	31.60	1502	1503	0.2 ± 0.0	0.1 ± 0.0
	32.39	1521		-	0.0 ± 0.0
	32.54	1525		-	0.0 ± 0.0
γ-Muurolene	32.94	1535	1526	0.7 ± 0.0	0.7 ± 0.0
δ-Cadinene	33.33	1544	1541	0.3 ± 0.0	0.3 ± 0.0
	34.31	1569		-	0.0 ± 0.0
Caryophyllene oxide	35.58	1601	1617	0.1 ± 0.0	0.1 ± 0.0
**Total identified compounds (%)**				99.8	99.6

Results are expressed as a percentage (%) of peak area ± standard deviation. Compounds are listed in order of elution from the HP-5MS column. The major compound is highlighted in bold. R.T.: retention time of the eluted compounds expressed in minutes. RI_exp_: experimental retention index calculated relative to n-alkane series standards (C7–C30) on the HP-5MS column. RI_lit_: literature retention index on a similar phase column [27].

**Table 5:** Determination of minimum inhibitory concentration (MIC) of *C. nardus* essential oil against the tested bacterial strains ^1^.

*C. nardus* Essential Oil Concentration (%)	Bacterial Strain	
	*Staphylococcus aureus*	*Escherichia coli*
1.25	+	+
0.625	+	+
0.313	+	−
0.156	−	−
0.078	−	−
0.039	−	−
0.020	−	−
0.010	−	−

^1^ Results are presented as the mean of three replicates. The symbol (+) indicates inhibition of bacterial growth, whereas (−) indicates no inhibition.

## Data Availability

The data are contained within the article.
